# Prognostic significance of ventricular function and late gadolinium enhancement on CMR in symptomatic patients with scleroderma

**DOI:** 10.1186/1532-429X-15-S1-P160

**Published:** 2013-01-30

**Authors:** Arun Natarajan, David Dobarro, Claire E Raphael, Arun J Baksi, Heiko Kindler, Raad Mohiaddin, Dudley Pennell, Benjamin Schreiber, Sanjay K Prasad

**Affiliations:** 1 CMR Unit, Royal Brompton Hospital, London, UK, London, UK; 2 Pulmonary hypertension unit, Royal Free Hospital, London, UK

## Background

Cardiac involvement is a leading cause of morbidity and premature mortality in patients with scleroderma. Identification of this offers the opportunity for earlier and more stratified therapeutic intervention. Published data on the prognostic significance of left and right ventricular impairment and myocardial fibrosis in this cohort are limited. The study objective was to determine the prevalence and prognostic significance of abnormalities on cardiovascular magnetic resonance (CMR) in patients with scleroderma who have breathlessness and/or other cardiac symptoms.

## Methods

This is a retrospective longitudinal study of 126 consecutive patients with confirmed scleroderma and cardiac symptoms, who had undergone CMR. Completed scans were available in 124 of these. All scans were performed at 1.5 Tesla (Siemens Sonata or Avanto). Thinned myocardium was defined as thickness <4mm, right ventricular hypertrophy defined as thickness>5mm. The presence of left ventricular (LV) or right ventricular (RV) dilatation was defined as an increase in indexed LV or RV volumes compared to previously published normal ranges. Late gadolinium enhancement (LGE) was defined as an area of clearly increased signal intensity confirmed on phase swapping. All scans were analysed by two independent operators. All cause mortality was determined from review of hospital records and the national summary care database. A Cox proportional hazards model was used to determine predictors of mortality (IBM SPSS 19, USA).

## Results

Demographic data and CMR findings are shown in Table 1. Mean age was 55 (range 19 to 82) years, 45% were male and 81% had at least one cardiovascular abnormality on the scan. Significant LV dysfunction (ejection fraction<45%) was evident in 12% of patients and reduction in RV ejection fraction in 20% of patients. Myocardial fibrosis by LGE was found in 21% of patients (Table 1). The number of patients with 1, 2 or 3 cardiovascular abnormalities on CMR were 13%, 13% and 10% respectively. In total, 46% of the patients had 4 or more abnormalities. There were 21 deaths during the follow-up period. CMR predictors of mortality were LV ejection fraction<45% (Hazard ratio [HR] 3.9, 95% confidence interval [CI] 1.52-9.84, P=0.004) and impaired RV ejection fraction (HR 2.6, 95%CI 1.04-6.38, P=0.04). The presence of LGE did not predict mortality (HR 1.05, 95%CI 0.34-3.16, P=0.94).

**Table 1:** Demographic data and CMR characteristics of 124 patients with symptomatic scleroderma.

Clinical and CMR characteristics	Number (of n=124)	Percentage (%)
Age	55	-
M:F	56:70	45% male, 55% female
Patients with at least one cardiac abnormality	100	83
Thinned LV myocardium	8	5
LV dilatation	24	19
LV wall motion abnormality	40	32
LVH	31	25
Raised LV mass index	23	19
Reduced LV long axis fx	37	30
LV EF<45%	15	12
RV dilatation	30	24
Reduction in RV EF	25	20
RVH	22	22
Reduced RV long axis function	22	17
Pericardial effusion	20	16
LA enlargement (>moderate)	27	22
RA enlargement (>moderate)	29	23
Dilated PA	45	36
Increased signal on T2-STIR	1	<1%
Late gadolinium enhancement	26	21
Patients with only 1 cardiovascular abnormality on CMR	16	13
Patients with 2 cardiovascular abnormalities on CMR	17	13
Patients with 4 or more cardiovascular abnormalities on CMR	57	46

CMR= cardiac magnetic resonance, LV=left ventricle, RV=right ventricle, EF=ejection fraction, MI=mass index, LVH=left ventricular hypertrophy, LA=left atrium, RA right atrium, PA=pulmonary artery, T2-STIR=Short Tau Inversion Recovery.

## Conclusions

This is, to the best of our knowledge, the largest cohort of patients with scleroderma to have undergone CMR. In this selective cohort, cardiovascular abnormalities on CMR are detectable in the majority (81%) of patients with scleroderma and breathlessness and/or other cardiac symptoms. Significant LV dysfunction was the strongest CMR predictor of mortality. Presence of LGE did not predict mortality.

## Funding

NA

